# Poor intraoperative visibility and postoperative astigmatism associated with trabecular micro-bypass stent for corneal dystrophy: A case report

**DOI:** 10.1097/MD.0000000000039211

**Published:** 2024-08-02

**Authors:** Suguru Nakagawa, Kiyoshi Ishii

**Affiliations:** a Department of Ophthalmology, Saitama Red Cross Hospital, Saitama, Japan.

**Keywords:** cataract surgery, minimally invasive glaucoma surgery, posterior polymorphous corneal dystrophy, trabecular micro-bypass stent

## Abstract

**Rationale:**

There are reports of safe cataract surgery in eyes with posterior polymorphous corneal dystrophy (PPCD); however, to our knowledge, there are no reports of minimally invasive glaucoma surgery (MIGS) in eyes with PPCD. Herein, we report a case of poor intraoperative visibility with gonioscopy, postoperative corneal edema, and corneal astigmatism in eyes with PPCD treated with trabecular micro-bypass stent combined with cataract surgery.

**Patient concerns/Diagnosis:**

A 78-year-old man was referred to our hospital for MIGS. He presented with bilateral corneal endothelial vesicular changes and band lesions. Endothelial cell density was 2983/2871 cells/mm^2^ (right/left eye), central corneal thickness was 581 μm/572 μm, best-corrected visual acuity values (Snellen equivalent) were 20/32 (right) and 20/100 (left), and corneal astigmatism was −2.7D in the right eye and −2.5D in the left eye.

**Intervensions/ Outcoms:**

After phacoemulsification and aspiration with intraocular lens implantation with a 2.4-mm corneal incision in both eyes, trabecular micro-bypass stents were inserted successfully despite the poor intraoperative visibility with gonioscopy. One week after surgery, the central corneal thickness was 614 μm/609 μm, and Descemet’s membrane folds and mild corneal edema were observed. Best-corrected decimal visual acuity was 20/40 for the right eye and 20/50 for the left eye. In the left eye, total corneal astigmatism increased from −2.5D to −5.5D. Corneal astigmatism and edema showed gradual improvement.

**Lessons:**

Although reports have shown that cataract surgery can be safely performed in eyes with PPCD, MIGS in eyes with PPCD may require caution regarding intraoperative visibility with gonioscopy and visual function in the early postoperative period.

## 1. Introduction

Posterior polymorphous corneal dystrophy (PPCD) is an autosomal-dominant inherited disorder of the corneal endothelium and Descemet’s membrane.^[1–3]^ The clinical presentation is highly variable, with reports of vision loss due to corneal opacity and astigmatism, although most cases are asymptomatic and are described as either nonprogressive or slowly progressive.^[4]^ One previous study reported a decrease in corneal endothelial cell density in PPCD eyes,^[5]^ whereas another reported a wide range of values (613–3405 cells/mm^2^).^[6]^ In younger patients, corneal steepening has also been reported.^[7,8]^ Corneal vesicle lesions are associated with corneal astigmatism. There are 2 reports of cataract surgery for PPCD, both of which reported that the surgery was performed safely.^[9,10]^ Savini et al^[10]^ reported no corneal edema and postoperative visual acuity of 20/20 on the day after surgery in 2 cases of cataract surgery for PPCD. However, Ayyildiz^[9]^ reported mild postoperative corneal edema and visual acuity of 20/63. Ayyildiz^[9]^ speculated that the reason for the poor visual acuity of 20/63 may have been due to mild corneal edema and surface irregularity unlike Savini et al.^[10]^

In this report, we describe a case of PPCD in which a micro-bypass stent was successfully inserted despite poor intraoperative visibility with gonioscopy. Postoperative corneal edema was observed, resulting in a greater than expected transient increase in corneal astigmatism in the early postoperative period.

## 2. Patient information

A 78-year-old man was referred to our hospital for minimally invasive glaucoma surgery (MIGS) in 2023.

## 3. Clinical findings

**Figure 1.**
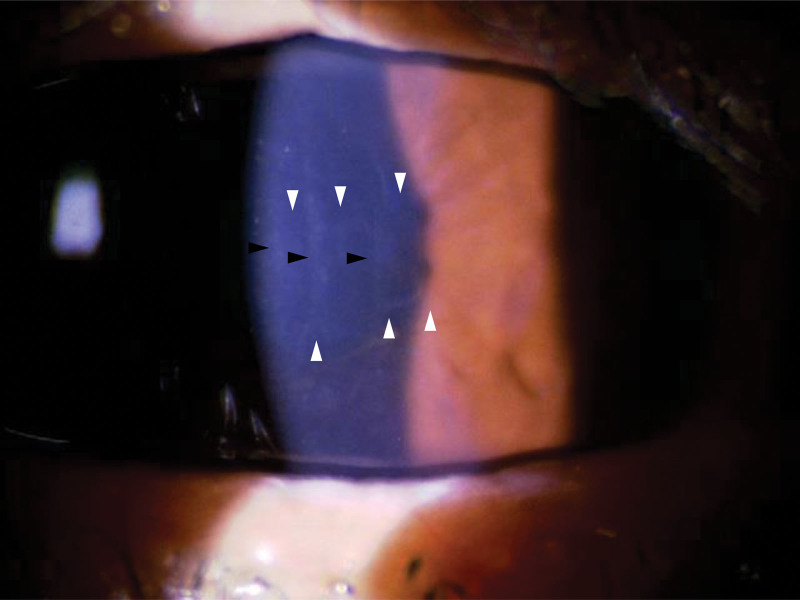
Corneal endothelial band lesion in the left eye observed 1 mo postoperatively. Horizontally extending corneal endothelial band lesions are observed (white arrowhead). The size and pattern of the band lesions did not change during follow-up. Descemet’s membrane folds (black arrowhead) and corneal edema were also found postoperatively.

**Figure 2.**
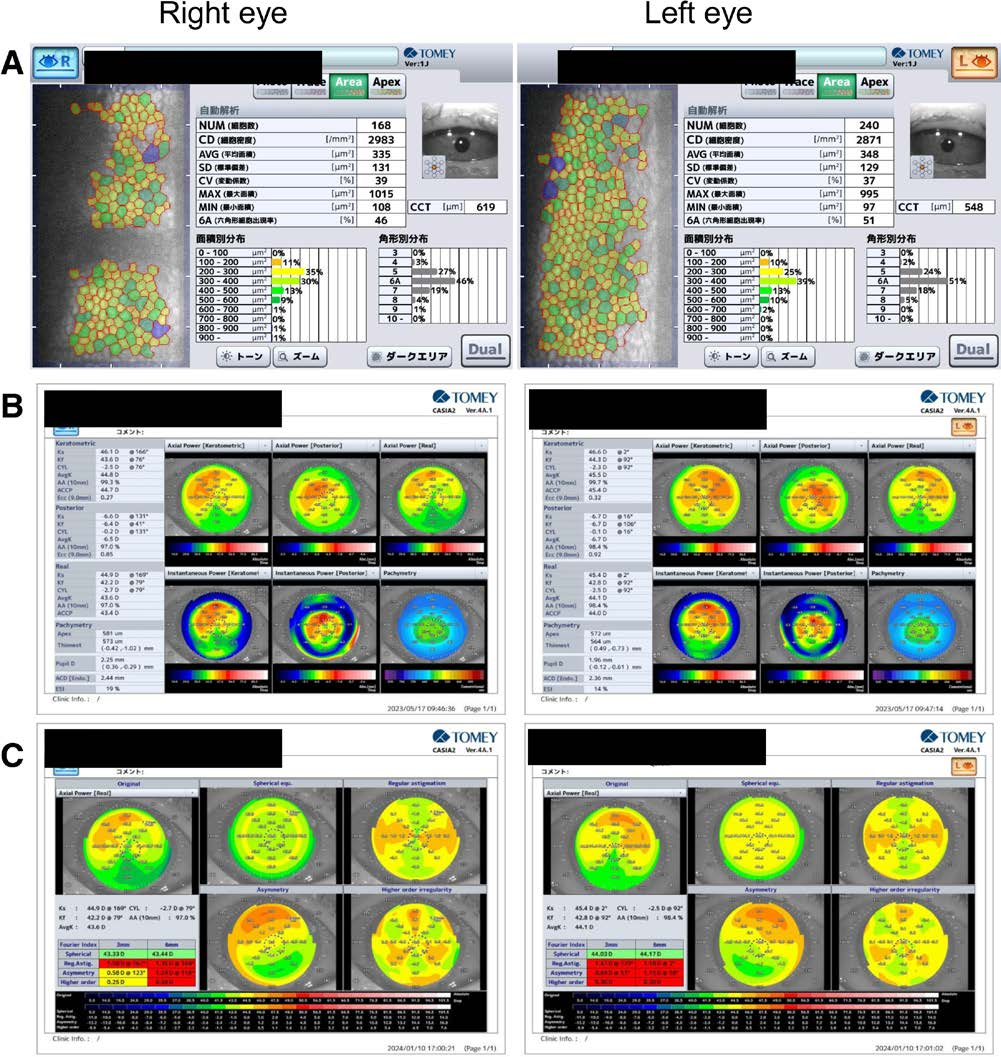
Preoperative specular microscope and corneal topography using anterior segment OCT. (A) Preoperative specular microscope. The lesion is the dark area. Endothelial cell density is preserved. (B) Preoperative corneal topography with anterior segment OCT. (C) Fourier analysis on corneal astigmatism. Preoperative regular astigmatism, asymmetric component, and higher-order aberrations were large in both eyes. OCT = optical coherence tomography.

In the preoperative examination, slit-lamp microscopy revealed corneal endothelial band lesions in both eyes (Fig. 1). Corneal endothelial cell densities were 2983 cells/mm^2^/2871 cells/mm^2^, central corneal thicknesses measured 581 μm/572 μm (Fig. 2), and the nuclear cataract grade was recorded as 3/3 (right/left eye). Best-corrected visual acuity (Snellen equivalent) was 20/32 and 20/100, and intraocular pressure (IOP) was 11 and 12 mm Hg under 2 glaucoma eye drops (2% carteolol hydrochloride and latanoprost) and 4 glaucoma drops (2% carteolol hydrochloride, latanoprost, brimonidine, brinzolamide), respectively (right/left eye).

## 4. Diagnostic assessment

The need for trabecular micro-bypass insertion in this case complied with the Japanese Ophthalmological Society’s criteria for the use of trabecular micro-bypass stents with phacoemulsification and aspiration. Inclusion criteria were mild to moderate open-angle glaucoma with cataract; IOP <25 mm Hg on antiglaucoma medications; and indication for cataract surgery with additional IOP reduction. The patient had mild glaucoma in the right eye and moderate glaucoma in the left eye, and both eyes met these inclusion criteria.

The manifest refractive cylinder was −4.5D/−4D, anterior and posterior corneal astigmatism (i.e., total corneal astigmatism) registered −2.7D/−2.5D, and Fourier analysis revealed that the asymmetric component was 0.58D/0.69D, whereas the higher-order aberration component was 0.25D/0.3D (right/left eye) (Table 1, Fig. 2).

**Table 1:** Visual acuity, astigmatism, and corneal thickness in this case

	Pre-op	1W	1M	2M	3M	4M
Right eye						
BCVA						
Snellen equivalent	20/32	20/40	20/50	20/32	20/32	20/32
Manifest refractive cylinder						
CYL (Diopter)	−4.5	−4.5	−4.5	−5	−5	−4.5
Total corneal astigmatism						
CYL (Diopter)	−2.7	−1.4	−2.6	−2.6	−1.3	−2.2
Fourier analysis of total corneal astigmatism						
Spherical (Diopter)	43.33	43	43.3	43.22	43.29	43.23
Reg. Astig. (Diopter)	1.58	1.14	1.6	1.58	1.04	1.63
Asymmetry (Diopter)	0.58	0.5	0.87	0.28	0.54	0.76
Higher order (Diopter)	0.25	0.49	0.37	0.39	0.36	0.44
Central corneal thickness						
μm	581	614	618	605	603	597
Left eye						
BCVA						
Snellen equivalent	20/100	20/50	20/100	20/63	20/32	20/63
Manifest refractive cylinder						
CYL (Diopter)	−4	−7	−8	−5	−5	−5
Total corneal astigmatism						
CYL (Diopter)	−2.5	−5.5	−4.6	−2	−2.8	−3.7
Fourier analysis of total corneal astigmatism						
Spherical (Diopter)	44.03	42.92	44.22	43.8	43.64	43.79
Reg. Astig. (Diopter)	1.47	3.86	2.49	1.1	1.35	1.86
Asymmetry (Diopter)	0.69	0.72	0.24	0.65	0.34	0.22
Higher order (Diopter)	0.3	0.83	0.32	0.19	0.22	0.27
Central corneal thickness						
μm	572	609	598	583	584	584

BCVA = best-corrected visual acuity, CYL = cylinder.

## 5. Therapeutic intervention

In both eyes, surgery was performed with a 2.4-mm upper temporal corneal incision after intra-anterior chamber anesthesia with 1% xylocaine. A viscoelastic substance (Healon V; Johnson & Johnson Surgical Vision, Santa Ana, CA) was used for anterior chamber formation, and phacoemulsification and intraocular lens implantation were performed by a skilled surgeon (KI) using up-to-date ultrasonic emulsification and aspiration equipment (CENTURION Vision System with ACTIVE SENTRY [Alcon Surgical, Fort Worth, TX]). Subsequently, 2 trabecular micro-bypass stents (iStent inject W [iSw]; Glaukos Corp. Laguna Hills, CA) were inserted, one through the lower region of the trabecular meshwork into Schlemm’s canal and the other through the lower nasal area of the trabecular meshwork into Schlemm’s canal. The first and second stents were 1 o’clock hours apart.

**Figure 3.**
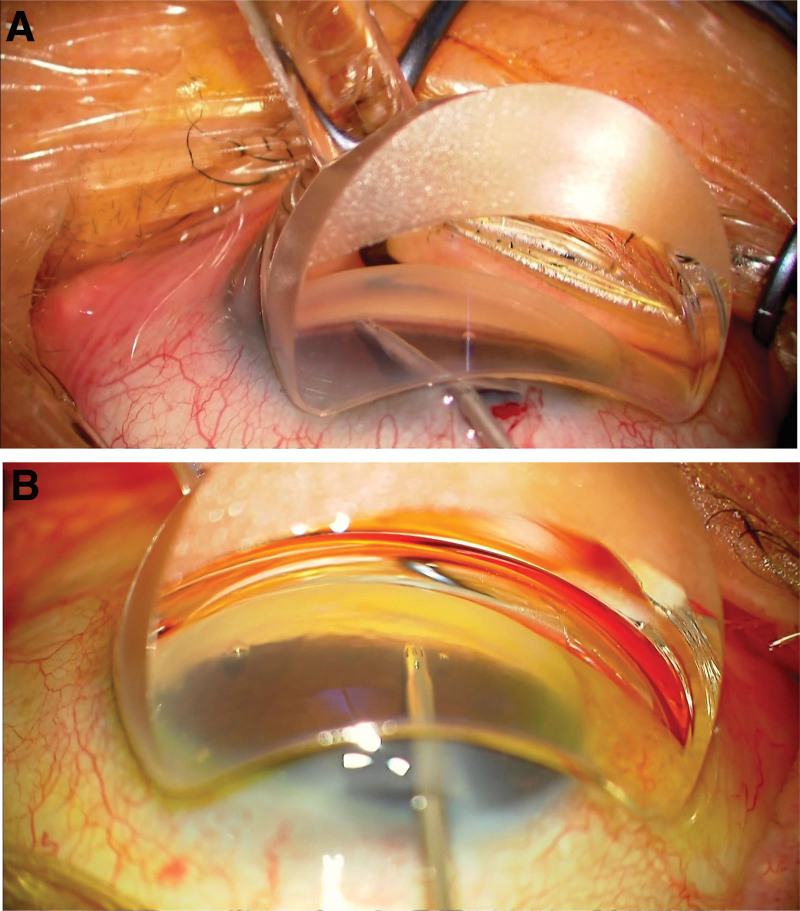
Intraoperative photographs of a normal case (A) and this case (B) at the time of micro-bypass stent insertion. In the normal case (A), the trabecular meshwork is clearly visible, but in this case (B), the visibility with gonioscopy is poor.

The trabecular meshwork was observed using gonioscopy (IPRISM; Glaucos Corp. Laguna Hills, CA). However, poorer visibility than usual was gained during surgery, making iSws insertion more difficult than usual (Fig. 3A is a typical case where good visibility with gonioscopy is gained, and Fig. 3B is this case where poor intraoperative visibility with gonioscopy is gained). The next day, the same surgery was performed on the other eye, and after phacoemulsification and aspiration with intraocular lens implantation with a 2.4-mm corneal incision, trabecular micro-bypass stents were inserted successfully despite the poor intraoperative visibility with gonioscopy.

## 6. Follow-up and outcomes

**Figure 4.**
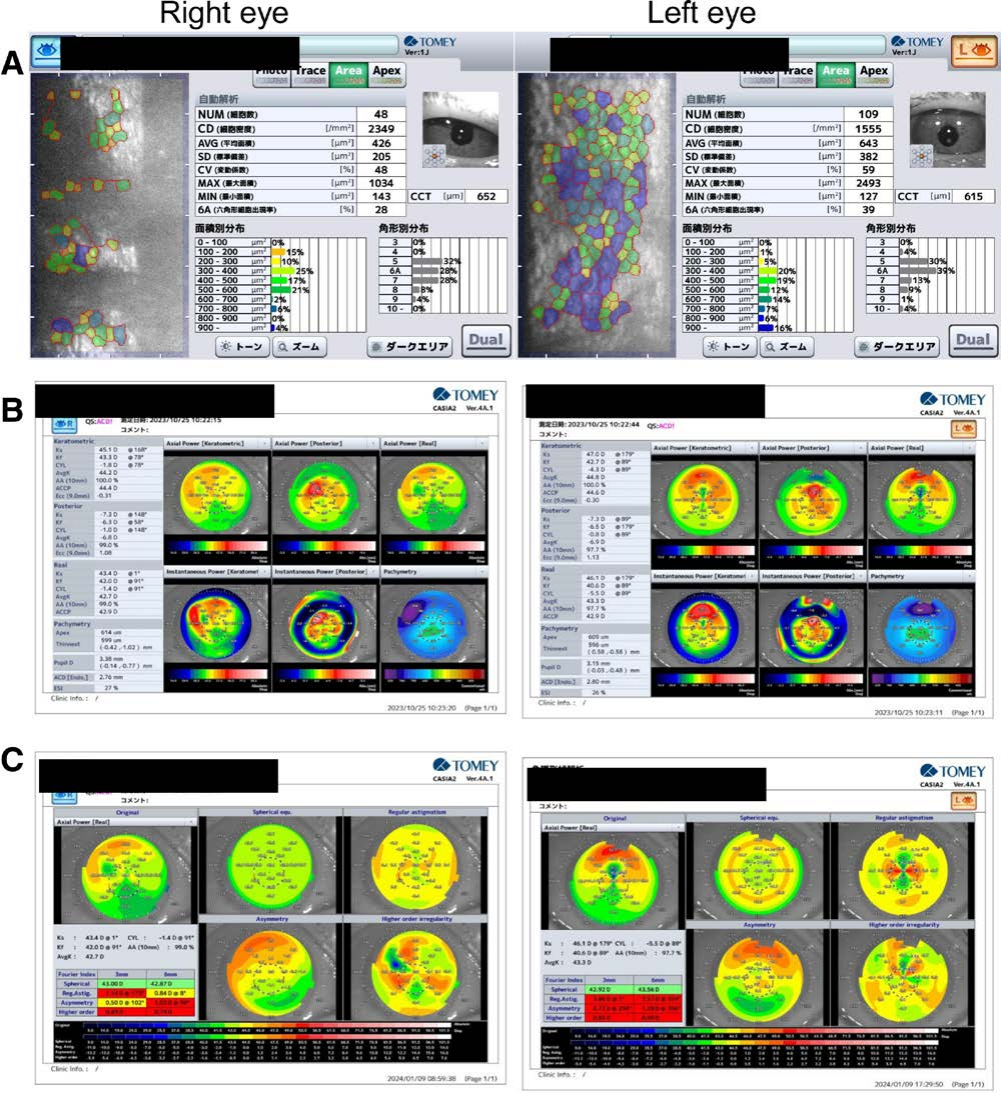
Specular microscope and corneal topography using anterior segment OCT 1 wk after surgery. (A) Specular microscopy 1 wk after surgery. (B) Corneal topography with anterior segment OCT 1 wk after surgery. (C) Fourier analysis on corneal astigmatism 1 wk after surgery. OCT = optical coherence tomography.

One week postoperatively, the endothelial cell counts were 2349 cells/mm^2^/1555 cells/mm^2^ (Fig. 4) and corneal thicknesses measured 614 μm/609 μm (right/left eye); mild corneal edema was observed (Fig. 4). Visual acuity 1 week after surgery was 20/40 for the right eye and 20/50 for the left eye. Astigmatism in the left eye increased in both manifest refractive cylinder and anterior and posterior corneal astigmatism (i.e., total corneal astigmatism) compared with preoperative levels. Manifest refractive cylinder increased from −4D to −7D and total corneal astigmatism increased from −2.5D to −5.5D. The IOP was 16.5 mm Hg in the right eye and 20.7 mm Hg in the left eye without any antiglaucoma eyedrop. Fourier analysis revealed a rise in regular astigmatism from 1.47D to 3.86D, a slight increase in the asymmetric component from 0.69D to 0.72D, and an escalation in the higher-order aberration component from 0.3D to 0.83D in the left eye (Table 1, Fig. 4).

Visual acuity 1 month postoperatively was 20/50 and 20/100, endothelial cell density was 2442 cells/mm^2^/1940 cells/mm^2^ (Table 1), corneal thickness measured 618 μm/598 μm, manifest refractive cylinder was −4.5D/−8D, and total corneal astigmatism was −2.6D/−4.6D (right/left eye). Two months postoperatively, the visual acuity was 20/32 and 20/63, endothelial cell density was not measurable/1556 cells/mm^2^, corneal thickness measured 605 μm/583 μm, the manifest refractive cylinder was −5D/−5D, and total corneal astigmatism was −2.6D/−2D (right/left eye) (Table 1).

**Figure 5.**
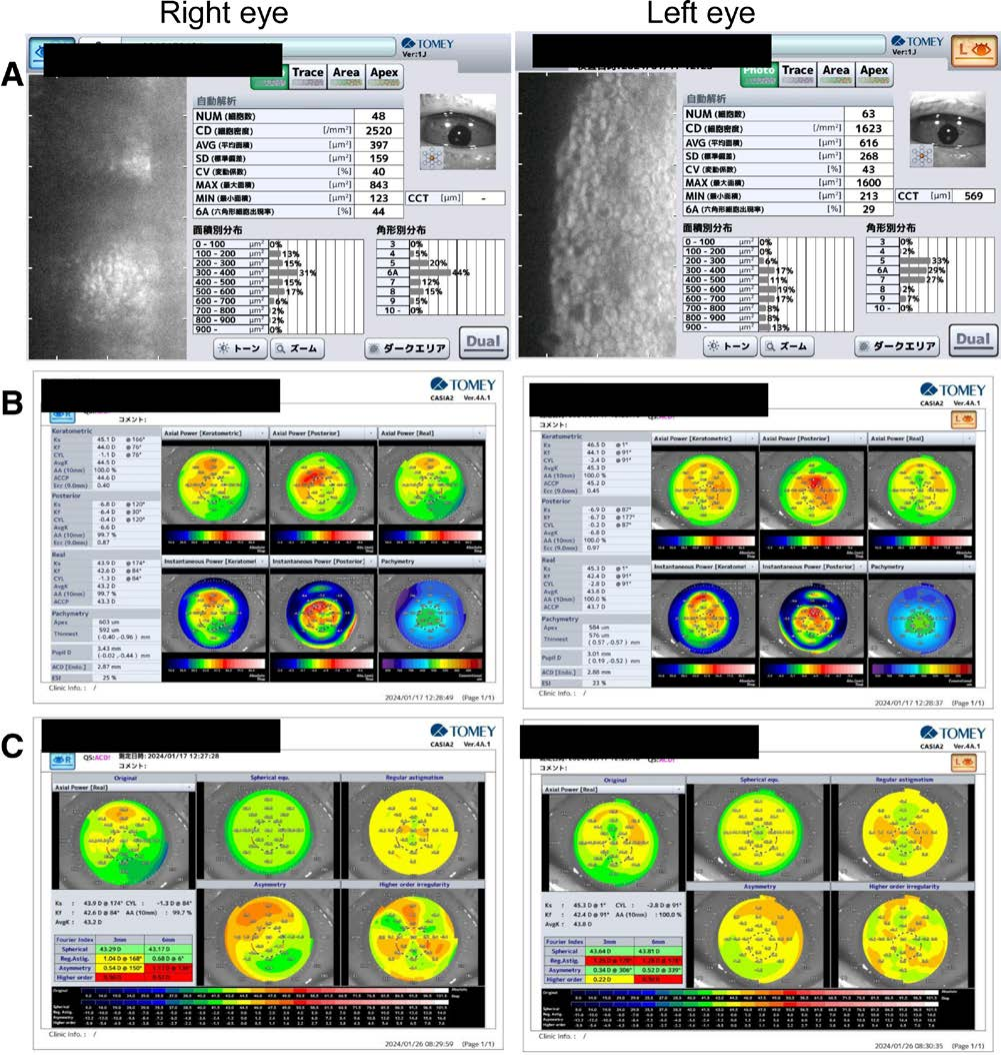
Specular microscope and corneal topography using anterior segment OCT 3 mo after surgery. (A) Specular microscopy 3 mo after surgery. (B) Corneal topography with anterior segment OCT 3 mo after surgery. (C) Fourier analysis on corneal astigmatism 3 mo after surgery. OCT = optical coherence tomography.

Three months postoperatively, the visual acuity was 20/32 and 20/32, endothelial cell density was 2520 cells/mm^2^/1623 cells/mm^2^ (Fig. 5), corneal thickness measured 603 μm/584 μm (Fig. 5), the manifest refractive cylinder was −5D/−5D, and total corneal astigmatism was −1.3D/−2.8D (right/left eye). Moreover, corneal edema in both eyes and astigmatism in the left eye showed gradual improvement (Fig. 5, Table 1). The size and pattern of the corneal endothelial band lesion did not change during follow-up.

## 7. Discussion

We report a case of poor intraoperative visibility with gonioscopy, postoperative corneal edema, and corneal astigmatism in eyes with PPCD treated by cataract surgery in combination with a trabecular micro-bypass stent.

Although there have been reports of safe cataract surgery in eyes with PPCD,^[9,10]^ to our knowledge, there are no reports of MIGS in eyes with PPCD. PPCD could theoretically increase the risk of postoperative complications for 2 reasons: the preoperative endothelial cell count is low and the behavior of the endothelial dystrophy foci is unpredictable. In the present case, the preoperative endothelial cell count was not low; however, the low intraoperative visibility and transient increase in postoperative corneal astigmatism in the early postoperative period were not expected.

Our observations suggest that even when preoperative endothelial cell counts are normal and cataract surgery is successfully performed as previously reported,^[9,10]^ it is difficult to insert the micro-bypass stents owing to limitations of gonioscopy (i.e., low visibility) in patients with corneas affected by PPCD, particularly during the surgery. Since cataract surgery is performed by observing the cataract from directly above the cornea, even if a small amount of corneal opacity is present, it will not cause any problem during surgery. However, during MIGS, the trabecular meshwork is observed obliquely to the cornea with gonioscopy; therefore, even a mild corneal lesion, opacity, or astigmatism in the center of the cornea may have a significant impact on visibility during gonioscopy. Patients may exhibit various issues after surgery, including Descemet’s membrane folds and unexpected corneal edema. It is important to consider these possibilities when performing MIGS and cataract surgery for PPCD.^[6]^

In the present study, despite the minimal surgical invasion of the corneal endothelium by employing a hard-shell technique with Healon V and utilization of the latest ultrasonic phacoemulsification and aspiration device by a skilled surgeon, intraoperative gonioscopy visibility was poor, and postoperative corneal edema and corneal astigmatism were greater than expected in the early postoperative period. Based on these findings, MIGS for PPCD may be challenging owing to the poor intraoperative visibility with gonioscopy even when cataract surgery for PPCD is successful. Moreover, visual function may deteriorate owing to greater postoperative corneal edema and astigmatism than expected in the early postoperative period. However, after a transient postoperative increase in astigmatism and corneal edema, most of the corneal changes and astigmatism appeared to return to baseline within 2 months postoperatively. Nevertheless, the lack of significant improvement in visual acuity after surgery thereafter may be due to mild corneal edema and corneal astigmatism due to PPCD. Additionally, considering the potential for postoperative changes in corneal astigmatism, a toric intraocular lens should probably not be inserted for PPCD. Despite intraoperative difficulties, the iStent inject W was inserted successfully, and the postoperative corneal edema and corneal irregular astigmatism showed improvement at follow-up.

Our analysis was limited to a 4-month follow-up, and a longer follow-up may yield different results.

In summary, MIGS in eyes with PPCD requires caution because it may be difficult to insert the micro-bypass stents owing to limitations of gonioscopy (i.e., low visibility) in patients with PPCD and corneal astigmatism may change more than expected after surgery.

## Acknowledgments

We thank Editage (www.editage.com) for English language editing.

## Author contributions

**Conceptualization:** Suguru Nakagawa, Kiyoshi Ishii.

**Data curation:** Suguru Nakagawa, Kiyoshi Ishii.

**Formal analysis:** Suguru Nakagawa, Kiyoshi Ishii.

**Investigation:** Suguru Nakagawa, Kiyoshi Ishii.

**Methodology:** Suguru Nakagawa, Kiyoshi Ishii.

**Writing – original draft:** Suguru Nakagawa.

**Writing – review & editing:** Suguru Nakagawa, Kiyoshi Ishii.

**Supervision:** Kiyoshi Ishii.
